# A Rare Case of Streptococcus Pneumoniae Causing Malignant Otitis Externa Complicated by Skull Base Osteomyelitis

**DOI:** 10.7759/cureus.25692

**Published:** 2022-06-06

**Authors:** Gennifer Wahbah Makhoul, Olivia Mobarakai, Umesh Manchandani, Neville Mobarakai

**Affiliations:** 1 Internal Medicine, Staten Island University Hospital, Staten Island, USA; 2 Internal Medicine, Touro College of Osteopathic Medicine, New York, USA; 3 Infectious Disease, Staten Island University Hospital, Staten Island, USA

**Keywords:** pseudomonas aeruginosa, malignant otitis externa, streptococcus pneumonia, skull base, osteomyelitis

## Abstract

Skull base osteomyelitis is an aggressive infection involving bones of the skull. It is a rare complication of malignant otitis externa, caused by the contiguous spread of the infection. Patients are mostly elderly with comorbidities that compromise immunity. It is atypical for *Streptococcus species *to be encountered in basilar skull osteomyelitis. Here we present the case of an 80-year-old male with multiple comorbidities including diabetes mellitus with a two-month history of right ear pain associated with occasional discharge and diminished hearing who was found to have bacteremia and basilar skull osteomyelitis with *Streptococcus pneumoniae *isolated from blood and otorrhea fluid cultures. This unusual presentation of *S. pneumoniae* related skull base osteomyelitis could be attributed to an undiagnosed pancreatic cancer at the time of presentation. Malignant otitis externa can progress into invasive disease in the head and neck; almost all cases tend to be caused by *Pseudomonas aeruginosa *but unusual cases, such as this, can be caused by *Streptococcus pneumoniae.*

## Introduction

Skull base osteomyelitis (SBO) is a life-threatening infection involving the temporal bone, central skull base, or atypical (sphenoid and occipital bone) skull base [1]. SBO was first described in 1959 by Meltzer and Kelemen in a case of temporal bone osteomyelitis, which itself is unusual, and most cases present in the setting of chronic and recurrent malignant otitis externa (MOE) [1-3].

SBO is most commonly seen in the setting of elderly diabetic male patients with a history of chronic malignant otitis externa (MOE) [4]. In 90% of MOE cases, *Pseudomonas aeruginosa *is the main causal organism as it is not part of the normal flora of the external auditory canal but is able to colonize after water exposure or minor trauma [5,6]. With a chronic infection of the external auditory canal, a common complication includes an extension of the disease from the auditory canal through the fissures of Santorini into the skull base [7]. In this case report, we explore an isolated case of *Streptococcus pneumoniae* skull base osteomyelitis in the setting of chronic malignant otitis externa in a diabetic elderly patient.

## Case presentation

An 80-year-old male with type 2 diabetes, atrial fibrillation, hypertension, and chronic obstructive pulmonary disease presented to the emergency department with a two-month history of right ear pain associated with occasional discharge and diminished hearing. The patient had visited the ENT department as an outpatient and had been using hydrocortisone-acetic acid drops and CSF otic Powder (chloramphenicol, sulfamethoxazole and an antifungal) without any relief. His right ear pain had progressively worsened the week prior to his arrival and had begun radiating to his right jaw and the back of his head; he also developed a fever and increased otorrhea. The patient had no history of head or ear trauma and denied the use of ear swabs, earbuds, and exposure to swimming pools. He denied any facial numbness, facial weakness, visual changes, nausea, vomiting, or recent travel. He is a former smoker, having quit over 30 years ago, and denied excessive alcohol or drug use. However, he had oxacillin-resistant *Staphylococcus aureus* bacteremia two years prior to this admission.

**Figure 1:**
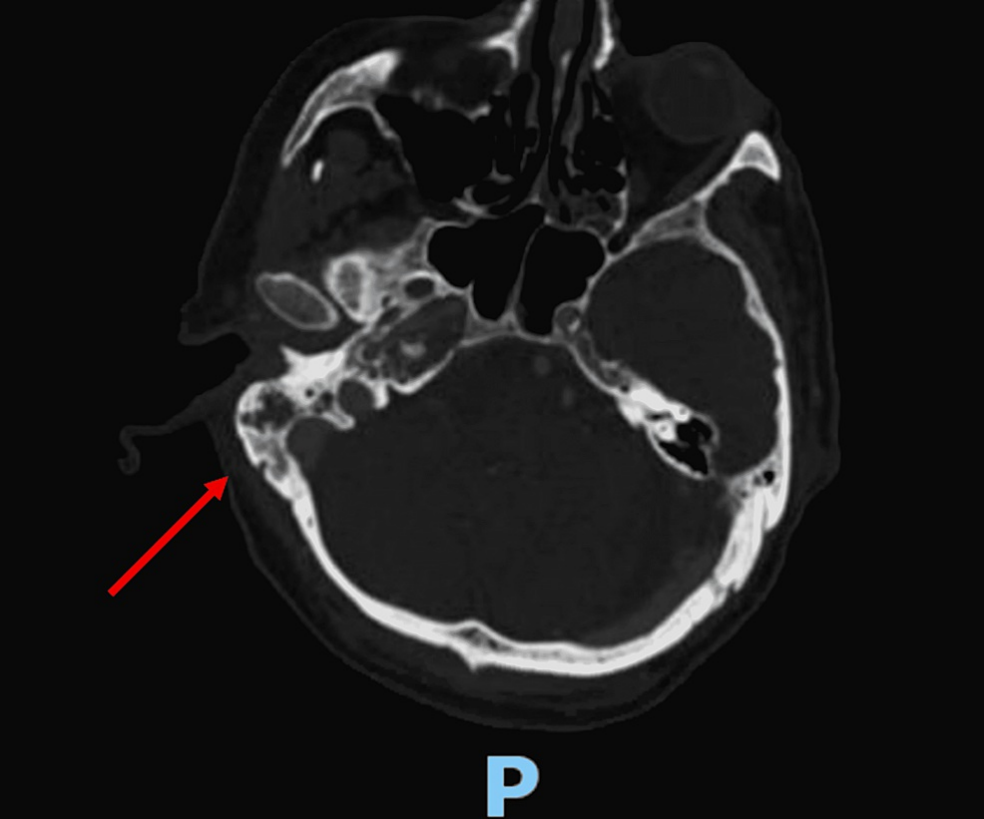
Computerized tomography scan of the head with intravenous contrast seen in bone window and axial view showing right otomastoiditis (red arrow)

**Figure 2:**
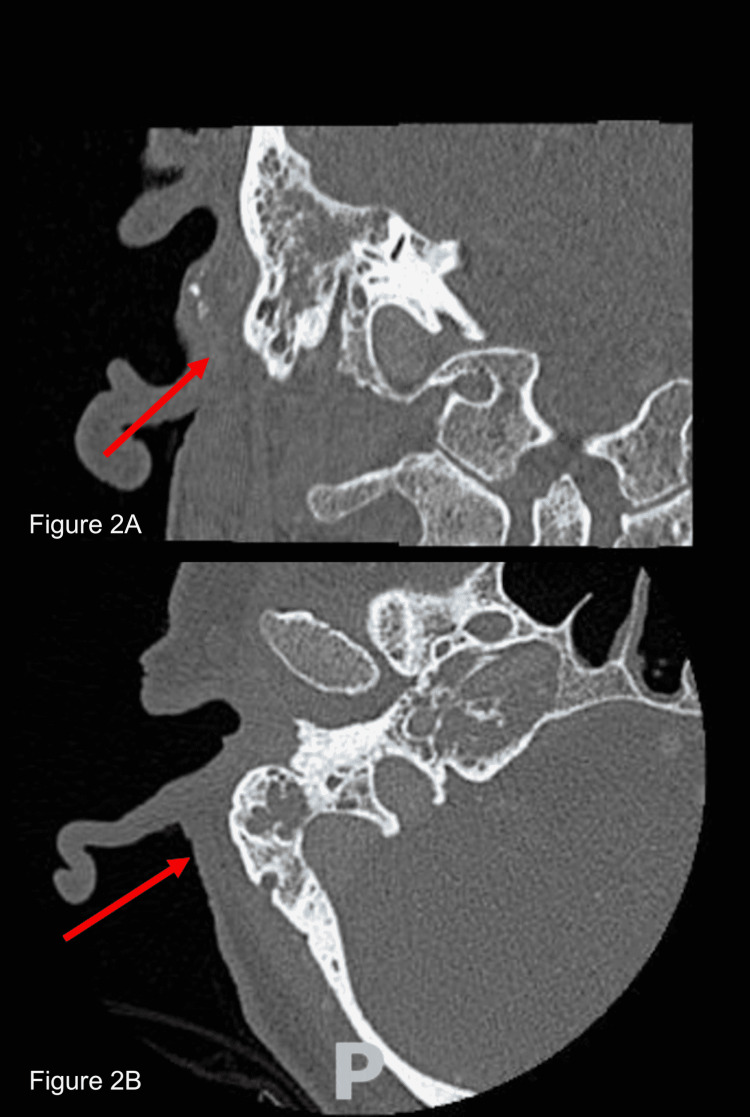
Computed tomography scan of the orbits with intravenous contrast (A) Coronal bone view; (B) axial bone view, both depicting right otomastoiditis with mastoid air cell septal erosion (red arrows).

Vital signs in the emergency department showed a fever of 38.2 °C, blood pressure 178/112 mm Hg, heart rate of 96 beats per minute, respiratory rate of 18 breaths per minute, and oxygen saturation of 99% on room air. His right external auditory canal was swollen and tender. The tympanic membrane was obscured by purulent fluid and granulation tissue and could not be visualized. ENT was consulted and performed bedside suction and irrigation. Ear and blood cultures were taken. The patient received ofloxacin ear drops and empiric intravenous (IV) antibiotic treatment was initiated with a combination of vancomycin and cefepime. Initial laboratory findings were remarkable only for hyperglycemia. Computed tomography (CT) of the orbits with IV contrast revealed right otomastoiditis with mastoid air cell septal erosion (Figures 1-2). Erosive changes involving the right petrous apex with dehiscence of the carotid canal were also seen, possibly representing superimposed SBO. 

The following day, initial blood cultures grew *Streptococcus pneumoniae*. His antibiotic regimen was adjusted to vancomycin, ceftriaxone, and levofloxacin. Cultures from the ear drainage grew *Streptococcus pneumoniae* and methicillin-resistant *Staphylococcus aureus* (MRSA). Transesophageal echocardiogram was performed and showed no valvular vegetations. Given the presence of pneumococcal bacteremia in the setting of concomitant osteomyelitis, diagnostic lumbar puncture was done to exclude meningeal involvement. The cerebrospinal fluid (CSF) sample analysis yielded zero white blood cells, mildly elevated protein levels, and normal glucose levels. There were no organisms on the gram stain and CSF fluid cultures were negative. Additional laboratory testing revealed an erythrocyte sedimentation rate of 79 mm/Hr and C-reactive protein of 30 mg/L. Magnetic resonance imaging of the brain with angiography was ordered to further assess the carotid canal involvement noted on CT. This was negative for arterial involvement, revealing only opacification of the right mastoid and middle ear erosion.

Repeat blood cultures performed 24 and 48 hours following the initial set of positive cultures showed no growth. As there was no evidence for MRSA bacteremia, it was determined that MRSA was a contaminant with the otorrhea fluid cultures. The antibiotic regimen was narrowed to ceftriaxone since the isolate was susceptible to penicillin and ceftriaxone with a minimal inhibitory concentration (MIC) value of 0.016 ug/mL using broth microdilution methodology. The patient was discharged home with a peripherally inserted central catheter and instructions to continue ceftriaxone IV every 12 hours for six weeks following his first set of negative blood cultures. On follow-up with infectious diseases clinic after the completion of his antibiotics, he had complete resolution of his right ear pain and discharge. Unfortunately, he presented to the hospital three months later with altered mental status and was diagnosed with a non-ST elevation myocardial infarction on arrival. Imaging of the head revealed acute bilateral cortical and right cerebellar infarcts. His hospital stay was further complicated by a pancreatic mass with possible metastasis to the liver. He eventually expired from multi-organ failure secondary to suspected metastatic pancreatic cancer. 

## Discussion

SBO is an aggressive infection involving any of the following bones compromising the skull: the temporal bone, central skull base, or atypical skull base (sphenoid and occipital bone) [1][5]. In most cases, SBO is associated with concomitant involvement of the mastoid process of the temporal bone, jugular vein, facial nerve, parotid gland, and temporomandibular joint [2]. SBO is a rare complication of MOE caused by the contiguous spread of the infection via the fissures of Santorini in the external auditory canal and subsequently through subfascial planes [3,5,6]. With the progression of the infection, granulation tissue grows and erosion of the skull base eventually occurs [7]. The causative pathogen of SBO can be from organisms also involved in MOE which in most cases is *Pseudomonas aeruginosa* [2].

The risk of developing SBO from MOE is based upon a patient’s age, comorbidities, and the presence of chronic inflammatory processes [3]. Common risk factors for SBO development include corticosteroids use, HIV infection, chronic inflammatory sphenoid disease, and most significantly poorly controlled diabetes mellitus [1,3,6,8]. In 2017, a retrospective review of various SBO cases showed that 24 out of 26 patients were diabetic [6]. Patients with severe immunodeficiencies including neutropenia, malnutrition, leukemia, critical illness requiring admission to the intensive care unit, sickle cell disease, and active treatment with chemotherapy have a higher risk of developing subsequent fungemia and bacteremia from MOE, increasing the risk of progression to SBO [1,9,10]. Elderly males, such as the patient mentioned in our case, are most at risk for SBO. However, younger individuals are also at increased risk when concomitant HIV infection is present [7,8,10].

Extension of the disease starts from an extracranial focus of infection, such as the external auditory tract, and progresses into deeper areas of the skull. The mechanism of spread arises from a focal MOE and with time the infection penetrates the skin, cartilage, and bone in the external ear [5]. Further extension inferior from the external auditory canal into the fissures of Santorini and through fascial or subfascial planes leads to fasciitis of the subtemporal area [5,7]. Granulation tissue forms in the temporal and sphenoid bones as the infection spreads to involve the mastoid process [7,10]. The infection progresses into the skull foramina with involvement of the facial nerve, but an extension to meningitis and brain abscesses is also possible [7]. 

*Pseudomonas aeruginosa* is the most common pathogen involved in SBO in the setting of a previous MOE, especially in elderly diabetic patients. Here we presented a rare case of mastoiditis and SBO caused by *Streptococcus pneumoniae* as a progression from MOE. *Streptococcus pneumoniae* is one of the most common bacterial causes of acute otitis media in children but is infrequently associated with ear infections in adults. Widespread use of pneumococcal conjugate vaccines has led to a dramatic decrease in pneumococcal infections [9]. *Streptococcus pneumoniae* has been associated with vertebral osteomyelitis in adults, with very few reported cases of acute mastoiditis [11]. Despite this, some cases of SBO caused by atypical organisms have been reported such as Streptococcus species, Staphylococcus aureus, and less commonly fungal organisms such as Aspergillus and Candida [4,8]. 

Given the mechanism of progression of this disease, patients who suffer from SBO are at increased risk of developing invasive disease of the brain. Various complications arise from SBO, given the location of the infection it can further develop neuropathies, soft tissue involvement of the cavernous sinuses, and leptomeningeal involvement [3]. Meningitis can be a common complication in isolated otitis media infection but not in otitis externa; in our case, the presence of concomitant bacteremia, as well as skull osteomyelitis, increases the likelihood of extension to meningitis, thus warranting the possible need for a lumbar puncture as part of the diagnostic workup in the case of SBO with bacteremia [4,10].

The above case describes an elderly male patient with *Streptococcus pneumoniae-*related SBO. A thorough workup showed an initial focus of infection to be a previously diagnosed and treated malignant otitis externa. To our knowledge, the presence of *Streptococcus pneumoniae* is unusual in cases of SBO secondary to MOE. Although no definitive risk factor can be ascertained, it is likely that the patient's comorbidities such as diabetes mellitus and an undiagnosed pancreatic cancer may have compromised immunity, thus increasing susceptibility to *Streptococcus pneumoniae* SBO. 

## Conclusions

Streptococcal* *MOE progressing into SBO is a rare health issue encountered in a diverse group of patients especially those with compromised immunity. Almost all cases of SBO tend to be caused by *Pseudomonas aeruginosa;* however other organisms may be implicated such as the case of our patient. *Streptococcus *species have been isolated as a cause of SBO; however, to our knowledge, no cases of *S. pneumoniae* have been reported. Healthcare providers should remain aware of the presence of these different bacterial species causing SBO and the presence of possible underlying factors causing a compromised immunity, such as malignancy in our patient. 
